# Exploring Medicinal Plants for Antimicrobial Activity and Synergistic Effects With Doxycycline Against Bacterial Species

**DOI:** 10.1155/2024/6238852

**Published:** 2024-10-03

**Authors:** Joel Frank Kenmeni, Ibrahim Sifi, Borel Ndezo Bisso, Prudence Ngalula Kayoka-Kabongo, Ulrich Joël Tsopmene, Jean Paul Dzoyem

**Affiliations:** ^1^Department of Biochemistry, Faculty of Science, University of Dschang, P.O. Box 67, Dschang, Cameroon; ^2^Department of Biology, Faculty of Sciences, University of Laghouat, Laghouat 03000, Algeria; ^3^Department of Agriculture and Animal Health, College of Agriculture and Environmental Sciences, University of South Africa, Florida, Pretoria, South Africa

## Abstract

Medicinal plants are rich sources of bioactive compounds with diverse pharmacological properties, including antimicrobial activities. This study aimed to assess the antibiofilm potential of methanol and ethanol extracts from nine selected medicinal plants, as well as their synergistic effects with doxycycline against *Bacillus* strains. Standard procedures were employed to determine the phytochemical composition, total phenolic, and flavonoid contents of the extracts. The antibacterial activity was evaluated using the broth microdilution method, while biofilm formation was assessed via the microtiter plate method. Antibiofilm activity was determined using the 3-(4,5-dimethyl-2-thiazolyl)-2,5-diphenyl-2H-tetrazolium-bromide (MTT) assay. Combination studies were conducted using the checkerboard microdilution method. All extracts contained phenols, flavonoids, steroids, triterpenes, and anthraquinones. The methanolic extract of *Psidium guajava* exhibited the highest total phenolic and flavonoid contents (90.48 ± 0.55 mg GAE/g), while the ethanolic extract of *Olax subscorpioidea* showed the highest flavonoid content (6.48 ± 0.33 mg QE/g). Ethanol extracts of *Eucalyptus globulus* and *Psidium guajava* and methanolic extract of *Syzygium jambos* demonstrated significant antibacterial activity against *Bacillus anthracis* 34F2 Sterne strains, with a MIC value of 64 *μ*g/mL. Biofilm formation in *Bacillus* strains was notably enhanced in the presence of glucose. The methanolic extract of *O. subscorpioides* exhibited the highest biofilm imbibition (85%), while *Picralima nitida* methanolic extract showed the most effective biofilm eradication (79%). The combination of *Solanum torvum* ethanol extract with doxycycline displayed synergistic effects against biofilm formation inhibition and eradication in all tested *Bacillus* strains. Taken together, *Solanum torvum* ethanol extract shows promise for developing new combination antibacterial therapies.

## 1. Introduction

The genus *Bacillus* is a Gram-positive rod-shaped, facultative anaerobic, spore-forming bacterium that causes several infections by eating contaminated animal products or inhaling spores [1]. Among *Bacillus* species, *Bacillus cereus* and *B. anthracis* are implicated in human infections such as anthrax arthritis, cutaneous infections, and foodborne diseases that represent important public health problems [1, 2]. *B. anthracis* is genetically monomorphic, closely related *to the Bacillus group* such as *B. cereus* and *B. thuringiensis*. *Bacillus cereus and B. anthracis* have high DNA sequence similarities but exhibit a spectrum of pathogen- and nonpathogenic-associated phenotypes [3]. The pathogenicity of *B. cereus* and *B. anthracis* is characterized by the formation of biofilms and the production of various enzymes, such as phospholipase C, sphingomyelinase, and proteases [4].

Biofilms are adherent microbial communities surrounded by an extracellular matrix composed of polysaccharides, extracellular DNA, lipids, proteins, and water [5, 6]. Chronic infections in human and animal hosts are associated with biofilm formation. In addition, biofilm formation can protect microbial pathogens against the host immune system and antimicrobial agents [4, 7]. Among the antibiotics used against *Bacillus* infections, *B. cereus* and *B. anthracis* are typically resistant to *β*-lactam antibiotics due to the production of *β*-lactamase enzymes but are more susceptible to the tetracycline class. However, resistance to this antibiotic class has been reported in some countries [4, 8]. Therefore, the development of new therapeutic options is urgently needed to control *Bacillus* infections.

Plants produce a wide variety of secondary metabolites such as alkaloids, phenols, flavonoids, saponins, terpenoids, steroids, quinones, and tannins [9–11]. These secondary metabolites are known as substances with tremendous biological potential (antimicrobial, antioxidant, inflammatory, antioxidant, antiquorum sensing, immunomodulatory, antidiabetic, anticancer, and other properties), with little or no toxic effects, which are vital in the management of many diseases and the basis for drug development [10, 12, 13].

*Chenopodium ambrosioides* L., also well known as *Dysphania ambrosioides*, belongs to the Amaranthaceae family and is widely distributed in Brazil and Cameroon [14]. It is used in traditional medicine to treat malaria, intestinal worms, hemorrhoids, diarrhea, cystitis, intestinal parasites, female infertility, liver diseases, and infections [14, 15]. *E. globulus* is a plant belonging to the Myrtaceae family. It is widely used in traditional medicine systems against several diseases, including asthma, bronchitis, and malaria [16]. *Mangifera indica* is a flowering plant that belongs to the family Anacardiaceae. The leaves of *M. indica* have traditionally been used for the treatment of several medical conditions, including gastrointestinal conditions, hemorrhages, hemoptysis, hemorrhoids, wounds, and ulcers [17]. *Olax subscorpioidea* is a woody shrub belonging to the family Olacaceae. In traditional medicine, it is used in the treatment of yellow fever, jaundice, venereal diseases, and guinea worm [18]. *P. guajava* is a tropical tree that belongs to the Myrtaceae family. The leaves of *P. guajava* are traditionally used for the treatment of diarrhea, diabetes, rheumatism, digestive problems, laryngitis, ulcers, and malaria [19, 20]. *P. nitida* (Apocynaceae) is a medicinal plant commonly used in traditional medicine to treat hypertension, jaundice, gastrointestinal disorders, and malaria [21]. *S. torvum* (Solanaceae) also known as *Eugenia jambos* L., *Jambosa jambos* Millsp., *Jambosa vulgaris* DC, and *Caryophyllus jambos* Stokes has been traditionally used to treat diabetes, hypertension, malaria, and tuberculosis [22–24]. *S. jambos* belonging to the Myrtaceae family is a native Asian tree that is commonly used in folk medicine in sub-Saharan Africa to treat hemorrhages, syphilis, leprosy, wounds, ulcers, and lung diseases [25]. *Thymus vulgaris* is a flowering plant of the Lamiaceae family and commonly used in folk medicine to treat various diseases, such as foodborne illnesses, skin infections, dermatitis, asthma, and bronchitis [26].

These selected medicinal plants have been shown to exhibit various pharmacological activities, such as antibacterial and antifungal properties against various microbial pathogens. Furthermore, the phytochemicals found in these medicinal plants, such as quercetin, vanillic acid, gallic acid, caffeic acid, chlorogenic acid, quinic acid, and thymol, have been reported to act synergistically with antimicrobials [27–30]. The combination of natural products with antibiotics has been reported to have more efficacy in fighting against various infections caused by microbial pathogens compared to monotherapies. Combination therapy improves drug absorption, improves the penetration into tissues and biofilm, and reduces side effects [31, 32].

However, studies on the antibiofilm activity of these selected medicinal plants against *Bacillus* species are lacking. Additionally, the combination effects of these medicinal plants with antibiotics have not been explored extensively. Therefore, this study was carried out to evaluate the potential of extracts from nine selected medicinal plants on the biofilm inhibition, as well as their combination effects with doxycycline against *Bacillus* strains.

## 2. Materials and Methods

### 2.1. Bacterial Strains and Medium

The two reference strains *Bacillus cereus* ATCC 33019 and *Bacillus anthracis* 34F2 Sterne used in this work were obtained from the Laboratory of Phytomedicine of the University of Pretoria. The bacterial strains were subcultured from the original culture stored at −80°C in Mueller Hinton broth (MHB, Dominique Dutscher SAS, France) containing 50% glycerol (v/v) and maintained on Mueller Hinton agar plates (MHA, Dominique Dutscher SAS, France) at 4°C and then aerobically grown at 37°C for 24 h for bioassays. The tryptic soya broth (TSB) medium was purchased from Dominique Dutscher SAS, France.

### 2.2. Chemicals

Doxycycline (CAS Number: 17086-28-1, purity: 97%) belongs to a class of drugs called tetracycline antibiotics and was purchased from Sigma-Aldrich. Dimethyl sulfoxide (DMSO, CAS Number: 67-68-5, purity: 99%), *p*-iodonitrotetrazolium chloride (INT, CAS Number: 146-68-9, purity: 98%), 3-(4,5-dimethythiazole-2-yl)-2,5-diphenyltetrazolium bromide (MTT, CAS Number: 298-93-1, purity: 98%), and Folin–Ciocalteu (CAS Number: 12111-13-6) reagents were purchased from Sigma-Aldrich.

### 2.3. Plant Material and Sample Preparation

Nine medicinal plants were investigated, leaves of *C. ambrosioides, E. globulus*, *M. indica*, *P. nitida*, *S. torvum*, *S. jambos*, and *T. vulgaris*; seeds of *O. subscorpoidea*; and stem of *P. guajava*. All plants were authenticated at the Cameroon National Herbarium, and voucher numbers were assigned (Table 1).

The plants were dried in the shade at room temperature, and then, 100 g of each dried powdered plant sample was macerated with 300 mL of methanol, and 100 g of the same plant sample was also macerated in 300 mL of 95% ethanol at room temperature for 48 h followed by filtration through Whatman No. 1 filter paper. The filtrates obtained were evaporated under reduced pressure at 60°C and 80°C using a rotary evaporator (Buchi R-200) to remove methanol and ethanol solvents, respectively. The extracts were kept at 4°C for future use.

### 2.4. Phytochemical Screening

The methanolic and ethanolic extracts of the studied medicinal plants were analyzed for alkaloids, steroids, triterpenes, flavonoids, phenols, tannins, saponins, anthraquinones, and anthocyanins using the method described by Harbone [62].

#### 2.4.1. Alkaloids

About 10 mg of extract was mixed with 3 mL of 50% hydrochloric acid (HCl) followed by the addition of a few drops of Mayer's reagent. The formation of precipitates indicated the positive presence of alkaloids.

#### 2.4.2. Triterpenes and Steroids

About 10 mg of extract was mixed with 3 mL of chloroform and 3 mL of acetic anhydride, and then, the mixture was cooled on ice for 3 min. Then, a few drops of concentrated sulfuric acid (H_2_SO_4_) were gently added. A red color formed in the lower chloroform layer revealed the presence of triterpenes, whereas successive appearance of blue, green, red, or orange colors revealed the presence of steroids.

#### 2.4.3. Flavonoids

About 3 mL of extract solution was mixed with 3 mL of 2% sodium hydroxide (NaOH). The change in yellow after the introduction of a few drops of concentrated HCl indicated the presence of flavonoids.

#### 2.4.4. Phenols

About 3 mL of extract solution was mixed with a few drops of 10% ferric chloride (FeCl_3_). The appearance of a green or blue color confirmed the presence of phenols.

#### 2.4.5. Tannins

About 10 mg of extract solution was added with 5 mL of water followed by boiling for 5 min. Then, 5 mL of 2% NaCl and 5 mL of 1% gelatin were added. The formation of precipitate showed the presence of tannins.

#### 2.4.6. Saponins

About 1 mL of extract solution was mixed with 5 mL of distilled water, and then, the mixture was agitated for 5 min. The formation of 1 cm of foam revealed the presence of saponins.

#### 2.4.7. Anthraquinones

About 10 mg of extract was dissolved in 4 mL of ether chloroform (1:1 v/v). Then, 4 mL of 10% NaOH was added. The formation of red color revealed the presence of quinones.

#### 2.4.8. Anthocyanins

About 10 mg was mixed with 5 mL of an aqueous solution of HCl (1% v/v). The presence of anthocyanins was marked by an orange color.

### 2.5. Determination of the Total Phenolic Content (TPC) and Total Flavonoid Content (TFC)

The Folin–Ciocalteu method as described by Sari et al. with some modifications was used to determine the TPC [63]. Briefly, 20 *μ*L of extract solution (0.0625 to 2 mg/mL) was mixed with 100 *μ*L of Folin–Ciocalteu reagent (10 times diluted) followed by incubation for 5 min. Then, 80 *μ*L of 20% Na_2_CO_3_ (w/v) was introduced into the mixture and incubated for 30 min. After incubation, the absorbance was measured at 765 nm using a microplate reader (SpectraMax 190, Molecular Devices, USA). The results were expressed as milligrams of gallic acid equivalents per gram of dry extract (mg GAE/g).

For the determination of the total flavonoid content, the aluminum chloride (AlCl_3_) colorimetric method was used as described by Sari et al. with minor modifications [63]. An aliquot of 100 *μ*L of extract (0.0625 to 2 mg/mL) was mixed with 50 *μ*L of 10% (w/v) AlCl_3_ and 50 *μ*L of potassium acetate (120 mM). After incubation of the mixture for 30 min at room temperature, the absorbance was measured at 415 nm using a microplate reader (SpectraMax 190, Molecular Devices, USA). The results were expressed as milligrams of quercetin equivalents per gram of dry extract (mg QE/g).

### 2.6. Antibacterial Activity of Methanolic and Ethanolic Extracts

The broth microdilution method was used to determine the minimum inhibitory concentration (MIC) and minimum bactericidal concentration (MBC) of extracts [64]. The microplate was filled with 100 *μ*L of bacterial inoculum (1.5 × 10^6^ CFU/mL), and then, 100 *μ*L of twofold serial dilutions of extracts (16 to 2048 *μ*g/mL) and doxycycline (4 to 512 *μ*g/mL) was added followed by incubation at 37°C for 24 h. Finally, 20 *μ*L of INT solution (0.2 mg/mL) was added and the microplate was incubated at 37°C for 30 min. The MIC was recorded as the lowest concentration of extracts or doxycycline that prevented the change in INT color from yellow to pink. For MBC determination, 50 *μ*L from each well exhibiting MIC and values above MIC was added to a microplate containing 150 *μ*L of MHB followed by incubation at 37°C for 48 h. The lowest concentration of extract that did not change the color INT was considered as MBC. The antibacterial activity of the extract was classified as follows: significant (MIC < 100 *μ*g/mL), moderate (100 MIC ≤ 625 *μ*g/mL), and low (MIC > 625 *μ*g/mL) [65].

### 2.7. Assessment of the Ability of *Bacillus* Strains to Form Biofilm

Biofilm formation in *Bacillus* strains was performed using the microtiter plate method as described by Ndezo et al. [27]. The 96-well flat-bottom sterile polystyrene microplate was filled with 200 *μ*L of bacterial inoculum (1.5 × 10^6^ CFU/mL) prepared in MHB, MHB plus 2% glucose, TSB, and TSB plus 2% glucose followed by incubation at 37°C for 24 h. Then, the medium in the wells was discharged followed by washing thrice with phosphate-buffered saline (PBS; pH 7.2) and the adherent cells were fixed with 200 *μ*L of methanol for 30 min. Next, the methanol in the wells was discharged followed by the addition of 200 *μ*L of safranin (1%) to stain the biofilm cells. Finally, 150 *μ*L of 95% ethanol was added to solubilize the stain and the absorbance (OD) was measured at wavelength 570 nm using a microplate reader. The wells filled with only MHB or MHB plus 2% glucose, and TSB or TSB plus 2% glucose were served as blank. The blank absorbance (ODc) was used to identify whether biofilm formation of *Bacillus* strains exists or not, and was calculated as follows: ODc = mean OD of negative control + 3 × standard deviation (SD) of negative control. The strain exhibiting OD < ODc, ODc < OD < 2 × ODc, 2 × ODc < OD 4 × ODc, and OD > 4 × ODc was considered nonbiofilm producer, weak biofilm producer, moderate biofilm producer, and strong biofilm producer, respectively.

### 2.8. Screening of Antibiofilm Activity of Extracts Against Inhibition of Biofilm Formation

Extracts were tested for their antibiofilm activity on inhibition of biofilm formation using the method of Bisso et al. [66]. An aliquot of 100 *μ*L of serial twofold extract dilutions (at concentrations of 2048 to 16 *μ*g/mL) and an aliquot of 100 *μ*L of bacterial inoculum (1.5 × 10^6^ CFU/mL) prepared in MHB plus 2% glucose were added to the flat-bottomed sterile polystyrene microplate of 96 wells followed by incubation at 37°C for 24 h. After incubation, the medium was discharged and 200 *μ*L of MTT solution (0.5 mg/mL) was added to the wells followed by incubation at 37°C for 4 h. The excess MTT solution was then removed, and 200 *μ*L of DMSO was added to the wells to dissolve the purple formazan crystals formed by the viable bacteria. Wells containing medium and bacteria were used as positive controls, while wells containing MHB broth without bacteria were used as blanks. The experiment was carried out three times. Absorbance was measured at wavelength 570 nm using a microplate reader, and these readings were then used to determine the percentage of biofilm inhibition: % biofilm inhibition = [1 − (OD_Test_ − OD_Blank_)/(OD_Control_ − OD_Blank_)] × 100.

### 2.9. Screening Antibiofilm Activity of Extracts Against Eradication of Biofilms

In this assay, the method described by Tokam et al. was used with slight modifications [67]. After 24 h of biofilm formation, the wells were washed three times with PBS, and then, the adherent cells were treated (at 37°C for 24 h) with 200 *μ*L of extract at a concentration of 2 MIC. Then, an MTT assay as described above was used to determine the percentage of biofilm eradication. The experiment was carried out three times.

### 2.10. Evaluation of the Synergistic Antibiofilm Effect of the Most Active Extracts in Combination With Doxycycline Against Inhibition of Biofilm Formation

For this assay, the most active extracts that showed a percentage of biofilm inhibition greater than 50% were combined with doxycycline against biofilm formation. The synergistic antibiofilm effect was investigated using the checkerboard method [68]. An aliquot of 50 *μ*L of extract (at concentration of 4096 *μ*g/mL) and doxycycline (at concentration of 128 *μ*g/mL) was serially diluted twice horizontally and vertically, respectively, in a 96-well flat-bottom polystyrene microplate followed by the addition of 100 *μ*L of bacterial strains (1.5 × 10^6^ CFU/mL) prepared in MHB plus 2% glucose. The plates were then incubated at 37°C for 24 h, and the MTT assay described above was used to determine the minimal biofilm inhibitory concentration (MBIC). The untreated wells served as the positive control, while the wells containing MHB supplemented with 2% glucose without bacteria were used as blanks. The percentage of biofilm inhibition was calculated as previously described, and the lowest concentration of extract or doxycycline that reduced biofilm metabolic activity by 100% was defined as MBIC. To determine the interaction between extract and doxycycline, the fractional inhibitory concentration index (FICI) was calculated using the formula: FICI = (MBIC of extract in the combination/MBIC of extract alone) + (MBIC of doxycycline in the combination/MBIC of doxycycline alone). The effect of the combination between the extract and doxycycline was judged by the values of the FICI, and the results were classified as synergy, additivity, indifference, and antagonism when FICI < 0.5, 0.5 < FICI < 1, 1 < FICI < 4, and FICI > 4, respectively. The experiment was carried out three times.

### 2.11. Evaluation of the Synergistic Antibiofilm Effect of the Most Active Extracts in Combination With Doxycycline Against the Eradication of Biofilm

To evaluate the potential synergistic antibiofilm effect of the most active extracts and doxycycline against the eradication of biofilm, the checkerboard method was used [68]. After 24 h of biofilm formation, the microplate was filled with 100 *μ*L of MHB plus 2% glucose, and 50 *μ*L of extract (at a concentration ranging from 64 to 4096 *μ*g/mL) and 50 *μ*L of doxycycline (at a concentration ranging from 0.125 to 128 *μ*g/mL) serial twofold dilutions were added, respectively, in horizontal and vertical orientation of the microplate, followed by incubation at 37°C for 24 h. The percentage of biofilm eradication was determined as described above, and the lowest concentration of extract or doxycycline that reduced biofilm metabolic activity by 100% was defined as the minimal biofilm eradication concentration (MBEC). FICI values were calculated and interpreted as previously described. The experiment was carried out three times.

### 2.12. Statistical Analysis

Statistical analysis was performed in GraphPad Prism version 8.0 using Fisher's least significant difference (LSD) and Student's *t*-test with two tails. Data obtained are presented as the mean ± SD of three independent experiments. The results were significantly different at *p* < 0.05.

## 3. Results and Discussion

### 3.1. Phytochemical Analysis and Extraction Yield

The results of the phytochemical analysis of the methanolic and ethanolic extracts of the medicinal plants studied are shown in Table 2. However, to extract these phytochemicals from plants, various solvents with different polarities are used. Among the different solvents used, methanol is known to be the most common solvents for the extraction of antioxidant compounds such as polyphenols of lower molecular weight, while ethanol is generally reported to improve the extraction yield of phytochemicals and is safe for human consumption [69].

Phytochemical screening showed that phenols, flavonoids, steroids, triterpenes, and anthraquinone were found in all extracts. These phytochemicals are known as compounds with several biological potential, such as antimicrobial, antioxidant, inflammatory, antioxidant, antiquorum sensing, immunomodulatory, antidiabetic, and anticancer properties [12]. These results corroborated those of Fankam et al., who reported the presence of phenols, flavonoids, steroids, triterpenes, and anthraquinone in the methanol extract of the *O. subscorpioidea* [70]. In addition, several studies reported the presence of these phytochemicals in the different plant extracts studied in this study [16, 24, 35].

Furthermore, phytochemical analysis showed that all extracts contained steroids, except the methanolic and ethanolic extracts of *O. subscorpioides*. Anthraquinones were present in all extracts except *M. indica* ethanolic extracts. The highest yields were recorded for the methanolic extracts of *E. globulus*, *S. jambos*, and *T. vulgaris*, and ethanolic extracts of *M. indica*, which were 13.45%, 10.39%, 10.22%, and 10.23%, respectively. Based on the yields obtained, methanol was the best extraction solvent, with 66.66% of medicinal plants showing a high yield percentage after methanol extraction.

### 3.2. TPC and TFC

**Figure 1:**
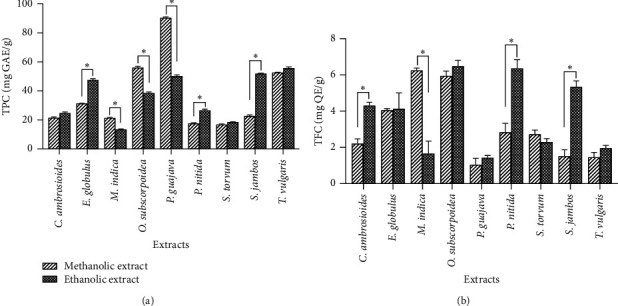
Total phenolic content (TPC) and total flavonoid content (TFC) of methanolic and ethanolic extracts of the medicinal plants studied. Data are presented as mean ± SD. Fisher's least significant difference (LSD) test was performed to compare the TPC and TFC of the extracts. ^∗^Significant difference at *p* < 0.05.

The results obtained from the determination of the TPC and the TFC of the extracts are plotted in Figure 1. The methanolic extract of *P. guajava* (90.48 ± 0.55 mg GAE/g) presented the highest TPC value, followed by the methanol extract of *O. subscorpioides* (56.1 ± 0.8855 mg GAE/g) and the ethanolic extract of *T. vulgaris* (55.78 ± 0.75 mg GAE/g), while the ethanolic extract of *M. indica* (13.53 ± 0.27 mg GAE/g) showed the lowest TPC value. Compared to the result of other studies, our TPC value in the methanolic extract of *P. guajava* is lower than those obtained by Majhi et al. (331.84 mg GAE/g) [71]. However, the TPC value in the ethanolic extract of *T. vulgaris* is almost similar to that of Vergun et al. (47.36 GAE/g) [72].

The highest TFC values were observed in the methanolic and ethanolic extracts of *O. subscorpioides* (5.95 ± 0.26 mg QE/g and 6.48 ± 0.33 mg QE/g, respectively) and the methanolic extracts of *P. nitida* (6.36 ± 0.48 mg QE/g) and *M. indica* (6.25 ± 0.13 mg QE/g), followed by the ethanolic extract of *S. jambos* (5.34 ± 0.33 mg QE/g), while the lowest TFC values were observed in the methanolic extract of *S. guajava* (1.04 ± 0.36 mg QE/g), *S. jambos* (1.51 ± 0.36 mg QE/g), and *T. vulgaris* (1.46 ± 0.26 mg QE/g). The TFC values obtained in the extracts from the previous studies are higher than our results [71, 72].

### 3.3. Antibacterial Activity

The antibacterial activity of the extracts is presented in Table 3. The ethanolic extracts of *E. globulus* and *P. guajava* and the methanolic extract of *S. jambos* showed significant antibacterial activity against the *B. anthracis* 34F2 Sterne strains with an MIC value of 64 *μ*g/mL. This significant antibacterial activity of plants could be attributed to the presence of vanillic acid in both extracts. Vanillic acid disrupts the cell membrane leading to bacterial cell death [73]. Additionally, the antibacterial activity of *P. guajava* could be attributed to the presence of quercetin in this plant. Previous studies have shown that quercetin has the ability to inhibit the synthesis of proteins and nucleic acids in bacterial cells. In addition, quercetin destroys the cell wall of bacteria, changes membrane permeability, and reduces the expression of virulence factors [74].

Ethanolic extracts of *S. torvum* exhibited significant antibacterial activity against strains of *B. cereus* ATCC 33019 with an MIC value of 64 *μ*g/mL. The presence of phytochemicals such as oleanolic acid in this plant could justify its antibacterial activity. Kim et al. have shown that oleanolic acid destroys the bacterial cell membrane [75].

The methanolic extracts of *E. globulus*, *P. nitida*, *P. guajava*, *S. torvum*, and *T. vulgaris* showed moderate antibacterial activity (with MIC values ranging from 128 to 512 *μ*g/mL) activity against the strains tested. Methanolic extracts of *M. indica* showed low antibacterial activity against the strains tested.

### 3.4. Biofilm Formation

**Figure 2:**
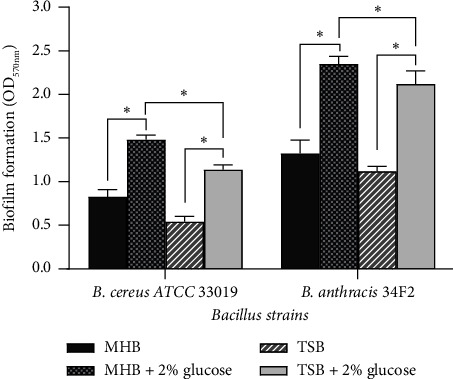
Influence of culture medium and presence of glucose on biofilm formation in *Bacillus* strains. ^∗^Significant difference at *p* < 0.05.

The ability of *Bacillus* strains to form biofilms in two different media in the presence of glucose is shown in Figure 2. The OD values of biofilm formation in two different cultures provide a carbon source for bacterial growth and metabolism, which could increase biofilm formation. These results corroborated those obtained in previous studies [76, 77].

In MHB supplemented with glucose, the biofilm formation of *B. cereus* ATCC 33019 and *B. anthracis* 34F2 was significantly higher than its biofilm formation in MHB, TSB, or TSB supplemented with glucose. Therefore, MHB supplemented with glucose was selected for antibiofilm assays. The composition of the medium strongly influences the ability of bacteria to form a biofilm. This has been confirmed in several studies, and the results have shown that TSB is sometimes better than MHB [78]. Furthermore, previous studies showed that medium plus glucose increased the ability of bacteria cells to form biofilms [78, 79]. Steixner et al. showed that supplementation of TSB or MHB with glucose increased the ability of bacteria to form biofilms and significantly increased the tolerance of biofilm cells to antibiotic [79].

### 3.5. Antibiofilm Activity

**Figure 3:**
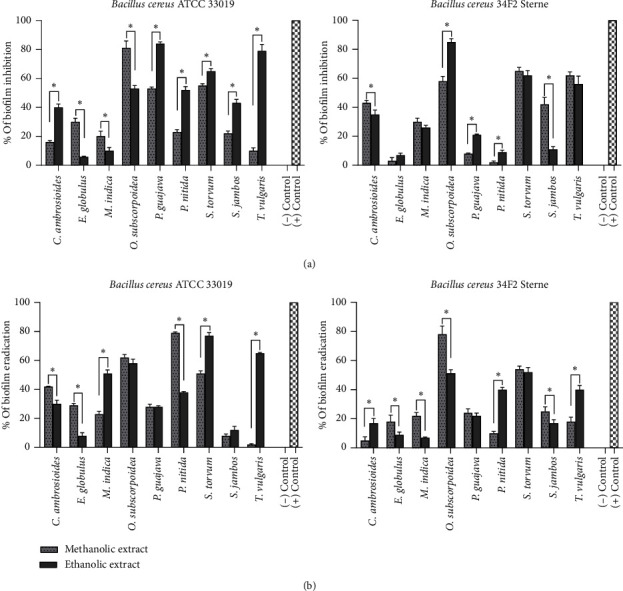
Percentage of (a) biofilm inhibition and (b) biofilm eradication of extracts in *Bacillus* strains. (−) control: treatment with DMSO only, (+) control: treatment with 1/2 MIC (8 *μ*g/mL) and 4 MIC (64 *μ*g/mL) of doxycycline for biofilm inhibition and eradication assays, respectively. Data are presented as mean ± SD. Student's *t*-test with two tails was performed to compare the percentage of inhibition and eradication of the biofilm of the methanolic and ethanolic extracts. ^∗^Significant difference at *p* < 0.05.

The resistance of *Bacillus* has been attributed to its ability to form biofilms on biotic and abiotic surfaces [80]. The inhibition of biofilm formation and the eradication of biofilms on surfaces covered by plant products may be significant in future techniques that prevent or treat biofilm-associated infections [81]. Hence, we evaluated the antibiofilm activity of plant extracts against *Bacillus* strains.  Figure 3 shows the antibiofilm activity of medicinal plant extracts at 1/2 MIC and 4 MIC on the inhibition of biofilm formation and eradication of biofilm in *Bacillus* strains, respectively.

The highest reduction of cell viability in the biofilm inhibition was observed in the methanolic and ethanolic extracts of *O. subscorpioides* (81% and 85%) and the ethanolic extracts of *P. guajava* (84%) and *T. vulgaris* (79%). The best reduction of cell viability in the biofilm eradication was detected in the methanolic extract of *P. nitida* (79%), the ethanolic extract of *S. torvum* (77%), and the methanolic extract of *O. subscorpioides* (78%). However, the reduction of cell viability in the inhibition and eradication of biofilm of other extracts ranged from 8% to 65% and from 2% to 65%, respectively. The methanolic and ethanolic extracts of *O. subscorpioides* and *S. torvum* showed biofilm inhibition and biofilm eradication percentages greater than 50% against all *Bacillus* strains tested. Therefore, these extracts were selected for combination studies. The presence of quercetin, rutin, and caffeic acid in extracts of *O. subscorpioides* and *P. guajava* could explain their higher antibiofilm. However, the presence of phytochemicals such as chlorogenic acid, vanillic acid, catechin, and ellagic acid in the extracts of *P. guajava* may also explain its antibiofilm activity. These phytochemicals suppress the activity of autoinducer-2-mediated cell–cell signaling responsible for cell-to-cell communication called quorum sensing, resulting in the inhibition of attachment and destruction of the structure of biofilms [82]. The antibiofilm activity of *T. vulgaris* could be due to the presence of thymol in this plant. Thymol is a monoterpene phenol and the main component found in the essential oils of *T. vulgaris*. Thymol inhibits extracellular matrix components such as polysaccharides; interferes with surface adhesion, thus preventing adhesion; and destroys the structure of biofilms by modulating the quorum sensing system [27]. Kuttinath, Murugan, and Rammohan reported the antibiofilm activity of *S. torvum* against methicillin-resistant *Staphylococcus aureus* [83].

### 3.6. Synergistic Antibiofilm Effect of Extracts and Doxycycline Against Inhibition of Biofilm Formation in *Bacillus* Strains

Another strategy used to overcome antibiotic resistance is the use of combination therapy to achieve synergistic effects [66]. The combined effects of the methanolic and ethanolic extracts of *O. subscorpioides* and *S. torvum* with doxycycline against inhibition of biofilm formation are presented in Table 4. On the basis of the FICI values, the combination of the methanolic extract of *S. torvum* with doxycycline and the combination of the ethanolic extract of *S. torvum* with doxycycline showed synergistic effects against inhibition of biofilm formation in all *Bacillus* strains tested. Furthermore, a synergistic effect (FICI = 0.188) was found in the combination of the methanolic extract of *O. subscorpioides* with doxycycline against inhibition of biofilm formation in strains of *B. anthracis* 34F2 Sterne. However, the ethanolic extract of *O. subscorpioides* combined with doxycycline exhibited indifference interactions against inhibition of biofilm formation in all *Bacillus* strains tested.

The interactions between extracts and doxycycline against biofilm eradication are shown in Table 5. Synergistic effects were observed in the combination of the ethanolic extract of *S. torvum* with doxycycline against the eradication of the biofilm in all *Bacillus* strains tested. Furthermore, a synergistic effect was found in the combination of methanolic *O. subscorpioides* with doxycycline against the eradication of the biofilm in *B. cereus* ATCC 33019.

These synergistic antibiofilm effects could be attributed to the inhibition of quorum sensing by the ethanolic extract of *S. torvum*, which enhances doxycycline activity. Khunbutsri et al. reported the synergistic activity of the *S. torvum* leaf extract and oxacillin against methicillin-resistant staphylococci isolated from dogs [55]. Taken together, our results indicated that the combination of *S. torvum* extract with doxycycline inhibited 100% of biofilm formation and eradicated 100% of mature biofilm. So, this combination could be useful in the fight against emerging drug-resistant *Bacillus* species.

## 4. Conclusion

Phytochemical analysis of the medicinal plant extracts studied revealed the presence of phenols, flavonoids, steroids, triterpenes, and anthraquinone in all extracts. The medicinal plants studied have promising antibacterial and antibiofilm activities against *Bacillus* strains. Synergistic antibiofilm effects observed in the combination of the ethanolic extract of *S. torvum* with doxycycline suggest that the extract of *S. torvum* could be a promising candidate for the development of new combination antibacterial therapy. However, more studies are needed to isolate and characterize the antibiofilm of the others compound responsible for its activity. In addition, antiquorum sensing activities are required to be conducted using other microbial strains for effective plant species. Cytotoxicity tests and in vivo bioactivity studies could be performed on plant species that exhibit significant antibiofilm activity.

## Figures and Tables

**Table 1:** Ethnopharmacological study of selected medicinal plants.

**Plant names (family)** **Voucher number**	**Part used**	**Ethnomedicinal uses**	**Pharmacological properties**	**Secondary metabolites isolated**
*Chenopodium ambrosioides* L. (Amaranthaceae) 33300/HNC	Leaves and stems	Malaria, intestinal worms, hemorrhoids, diarrhea, cystitis, intestinal parasites, female infertility, liver diseases, and infections [14, 33, 34]	Antiarthritic, amoebicidal, anthelmintic, antibacterial, antifungal, anticancer, antidiabetic, and antidiarrheal [14, 15, 35]	1-Piperoylpiperidine, octadecanoic acid, scopoletin, stigmasterol, *β*-sitosterol [36] 1,2-Benzopyrone, kaempferol, kaempferol 3-*O*-*α*-L-^1^C_4_ rhamnopyranoside (afzelin), kaempferol 3-*O*-*α*-L-^1^C_4_ rhamnosyl-(1‴ ⟶ 2″)-*β*-D-^4^C_1_-xylopyranoside, and kaempferol 7-O-*α*-L-1C4–rhamnopyranoside [37]

*Eucalyptus globulus* Labill. (Myrtaceae) N/A	Leaves	Asthma, bronchitis, malaria, and urinary and skin diseases [16, 38]	Antibacterial, antifungal, antiquorum sensing, anthelmintic, antidiabetic, anti-inflammatory, and antioxidant [39, 40] Antimalarial [16] Antiquorum sensing [41]	3,4-Dihydroxybenzoic acid (protocatechuic acid), *p*-coumaric acid, vanillic acid, avicularin, guaijaverin, and astragalin [40] (1,8-cineole), *α*-pinene, and (−)-globulol [41]

*Mangifera indica* L. (Anacardiaceae) 1846/HNC	Leaves, seed kernel	Cough, hiccup, hyperdipsia, hemorrhages, hemoptysis, hemorrhoids, wounds, ulcers, diarrhea, dysentery, and pharyngoplasty [17]	Anticancer, antioxidant, anti-inflammatory, antimicrobial, and immunomodulatory [42, 43]	Gallic acid, caffeic acid, rutin, and penta-O-galloyl-*β*-d-glucose [43]

*Olax subscorpioidea* Oliv. (Olacaceae) 3528/SRFK	Fruits	Constipation, yellow fever, jaundice, venereal diseases, and guinea worm [18]	Anthelmintic, analgesic, antiarthritic, antidepressant, antihyperglycemic, anti-inflammatory, antioxidant, antimalarial, and antimicrobial [44, 45]	Rutin, morin, quercetin, and caffeic acid [46] n-Hexadecanoic acid, 7,10,13-hexadecatrienoic acid, methyl ester, 9,17-octadecadienal, (Z), 9,12-octadecadienoic acid (Z,Z),octadecanoic acid, squalene, nonacosane, and hentriacontane [47]

*Psidium guajava* L. (Myrtaceae) 2884/SFRK	Leaves, barks	Diarrhea, diabetes, rheumatism, digestive problems, laryngitis, ulcers, malaria, cough, and bacterial infections [19, 20]	Antidiabetic, anticancer, antidiarrheal, hepatoprotective, antioxidant, anti-inflammatory, antiestrogenic, and antibacterial [48–50]	Chlorogenic acid, rutin, vanillic acid, quercetin, *p*-hydroxyl benzoic acid, syringic acid, caffeic acid, kaempferol, catechin, myricetin, isoquercetin, and apigenin [51] Reynoutria, guaijaverin, Morin, catechin, ellagic acid, guavinoside B, acetophenone, quercetin, (2,6-dihydroxy-3-methyl-4-O-(6″-O-galloyl-*β*-D-glucopyranosyl)-benzophenone), guavinoside C, and ellagitannin [52, 53]

*Picralima nitida* (Stapf) (Apocynaceae) 1942/SRFK	Leaves, stem bark	Fever, pain, hypertension, jaundice, gastrointestinal disorders, and malaria [21]	Antioxidant, antidiabetic, hypotensive, antiplasmodial, antimicrobial, antiulcer, and antitumorigenic [21, 54]	Dodecanoic acid, 9 octadecanoic acid, n-hexadecanoic acid [21]

*Solanum torvum* Swartz. (Solanaceae) 1651/SFR	Leaves, fruits	Diabetes, hypertension, malaria, and tuberculosis [23, 24]	Antibiofilm, antioxidant, cardiovascular, immunomodulatory, nephroprotective, antibacterial, and antifungal [22–24]	Hexadecanoic acid and 9,12,15-octadecatrienoic acid [55] Betulinic acid, 3-oxo-friedelan-20*α*-oic acid, sitosterol-3-*β*-D-glucopyranoside, and oleanolic acid [22]

*Syzygium jambos* L. Alston. (Myrtaceae) 30458/HNC	Leaves	Hemorrhages, syphilis, leprosy, wounds, ulcers, and lung diseases [25]	Antioxidant, antimicrobial, anti-inflammatory, anticancer, analgesic, antihyperglycemic, and antimalarial [25, 56, 57]	(E)‐caryophyllene, n‐heneicosane, *α*‐humulene, and thujopsan−2‐*α*‐ol [58]

*Thymus vulgaris* L. (Lamiaceae) 25746/HNC	Leaves and stems	Skin infections, acne, oily, dermatitis, foodborne illnesses, asthma, bronchitis, whooping cough, pharyngitis, cystitis, digestive system disorders, rheumatism, and arthritis [26]	Anticancer, immunomodulatory, antioxidant, neuroprotective, antidiabetic, antimicrobial, cardioprotective, nephroprotective, antiaging, hepatoprotective, anti-inflammatory, antiallergic, antidepressant, and antiviral [26, 59]	Quinic acid, rosmarinic acid, caffeic acid, ferulic acid, linalool, thymol, carvacrol, geraniol, *β*-caryophyllene, apigenin, and luteolin [26, 60, 61]

Abbreviation: NA = not available.

**Table 2:** Phytochemical screening and extraction yield of methanolic and ethanolic extracts from medicinal plants studied.

**Plants**	**Solvent**	**Phytochemical groups**									**Extraction yield (%)**
		**Alk**	**Phe**	**Fla**	**Ste**	**Tri**	**Tan**	**Sap**	**Ant**	**Ath**	
*C. ambrosioides*	M	−	+	+	+	+	−	−	−	+	7.72
	E	−	+	+	+	+	−	+	+	+	4.22

*E. globulus*	M	+	+	+	+	+	+	+	−	+	13.45
	E	+	+	+	+	+	+	+	−	+	9.71

*M. indica*	M	−	+	+	+	+	−	−	−	+	6.93
	E	+	+	+	+	+	−	+	−	−	10.23

*O. subscorpioides*	M	−	+	+	−	+	−	+	+	+	5.26
	E	−	+	+	−	+	−	+	−	+	4.41

*P. guajava*	M	+	+	+	+	+	−	−	+	+	3.19
	E	+	+	+	+	+	−	−	+	+	3.74

*P. nitida*	M	+	+	+	+	+	+	−	−	+	4.62
	E	+	+	+	+	+	+	−	−	+	5.59

*S. torvum*	M	−	+	+	+	+	−	−	−	+	7.62
	E	+	+	+	+	+	−	+	−	+	5.83

*S. jambos*	M	+	+	+	+	+	−	−	−	+	10.39
	E	+	+	+	+	+	−	−	+	+	5.83

*T. vulgaris*	M	+	+	+	+	+	+	−	−	+	10.22
	E	+	+	+	+	+	−	+	−	+	6.64

Abbreviations: Alk = alkaloids, Ant = anthocyanins, Ath = anthraquinones, E = ethanolic extract, Fla = flavonoids, M = methanolic extract, Phe = phenols, Sap = saponins, Ste = steroids, Tan = tannins, Tri = triterpenes.

*Note:* − = absence of phytochemicals, + =  presence of phytochemicals.

**Table 3:** MIC (*μ*g/mL) and MBC (*μ*g/mL) of extracts against *Bacillus* strains.

		**Strains**			
		***B. cereus* ATCC 33019**		***B. anthracis* 34F2**	
Antibiotic					

Doxycycline	MIC	16		16	
	MBC	32		32	

Plants		Methanolic extract	Ethanolic extract	Methanolic extract	Ethanolic extract

*C. ambrosioides*	MIC	1024	>1024	>1024	>1024
	MBC	1024	>1024	>1024	>1024

*E. globulus*	MIC	512	256	64	64
	MBC	>1024	>1024	512	512

*M. indica*	MIC	1024	1024	512	512
	MBC	>1024	>1024	>1024	>1024

*O. subscorpioides*	MIC	512	1024	1024	1024
	MBC	>1024	>1024	>1024	>1024

*P. guajava*	MIC	512	512	64	64
	MBC	>1024	1024	1024	1024

*P. nitida*	MIC	128	128	256	256
	MBC	>1024	512	>1024	>1024

*S. torvum*	MIC	512	64	128	128
	MBC	>1024	256	1024	1024

*S. jambos*	MIC	128	256	256	256
	MBC	1024	1024	1024	1024

*T. vulgaris*	MIC	512	256	128	128
	MBC	>1024	1024	>1024	>1024

Abbreviations: MBC = minimum bactericidal concentration (*μ*g/mL), MIC = minimum inhibitory concentration (*μ*g/mL).

**Table 4:** Combined effects of most active extracts with doxycycline against inhibition of biofilm in *Bacillus* strains.

**Strains**	**MBIC alone**		**MBIC in combination**		**FIC**		**FICI (antibiotic + extract)**	**Interaction**
	**DO**	**StM**	**DO**	**StM**	**DO**	**StM**	**(DO + StM)**	
*B. cereus*	8	128	0.625	16	0.078	0.125	0.203	Synergy
*B. anthracis*	16	256	0.5	64	0.031	0.250	0.281	Synergy

	**DO**	**StE**	**DO**	**StE**	**DO**	**StE**	**(DO + StE)**	

*B. cereus*	8	512	0.25	512	0.016	0.250	0.266	Synergy
*B. anthracis*	16	2048	0.625	256	0.039	0.125	0.164	Synergy

	**DO**	**OsM**	**DO**	**OsM**	**DO**	**OsM**	**(DO + OsM)**	

*B. cereus*	8	128	0.25	128	0.031	1	1.031	Indifference
*B. anthracis*	16	256	32	16	0.125	0.063	0.188	Synergy

	**DO**	**OsE**	**DO**	**OsE**	**DO**	**OsE**	**(DO + OsE)**	

*B. cereus*	8	128	0.125	128	0.016	1	1.016	Indifference
*B. anthracis*	16	256	0.625	512	0.039	2	2.039	Indifference

Abbreviations: DO = doxycycline, FIC = fractional inhibitory concentration, FICI = fractional inhibitory concentration index, MBIC = minimal biofilm inhibitory concentration (*μ*g/mL), OsE = ethanolic extract of *O. subscorpioides*, OsM = methanolic extract of *O. subscorpioides*, StE = ethanolic extract of *S. torvum*, StM = methanolic extract of *S. torvum*.

**Table 5:** Combined effects of most active extracts with doxycycline against eradication of biofilm in *Bacillus* strains.

**Strains**	**MBEC alone**		**MBEC in combination**		**FIC**		**FICI (antibiotic + extract)**	**Interaction**
	**DO**	**StM**	**DO**	**StM**	**DO**	**StM**	**(DO + StM)**	
*B. cereus*	16	256	32	128	2	0.5	2.5	Indifference
*B. anthracis*	64	2048	32	128	0.5	0.063	0.563	Additivity

	**DO**	**StE**	**DO**	**StE**	**DO**	**StE**	**(DO + StE)**	

*B. cereus*	16	512	2	128	0.125	0.250	0.375	Synergy
*B. anthracis*	64	2048	16	128	0.250	0.063	0.313	Synergy

	**DO**	**OsM**	**DO**	**OsM**	**DO**	**OsM**	**(DO + OsM)**	

*B. cereus*	16	128	4	128	0.250	1	1.25	Indifference
*B. anthracis*	64	512	2	256	0.031	0.5	0.531	Additivity

	**DO**	**OsE**	**DO**	**OsE**	**DO**	**OsE**	**(DO + OsE)**	

*B. cereus*	16	512	4	128	0.25	0.25	0.5	Synergy
*B. anthracis*	64	512	8	1024	0.125	2	2.125	Indifference

Abbreviations: DO = doxycycline, FIC = fractional inhibitory concentration, FICI = fractional inhibitory concentration index, MBEC = minimal biofilm eradication concentration (*μ*g/mL), OsE = ethanolic extract of *O. subscorpioides*, OsM = methanolic of *O. subscorpioides*, StE = ethanolic extract of *S. torvum*, StM = methanolic extract of *S. torvum*.

## Data Availability

The data used to support the findings of this study are available upon reasonable request from the corresponding author.
